# Cauliflower Leave, an Agricultural Waste Biomass Adsorbent, and Its Application for the Removal of MB Dye from Aqueous Solution: Equilibrium, Kinetics, and Thermodynamic Studies

**DOI:** 10.1155/2016/8252354

**Published:** 2016-11-15

**Authors:** Seraj Anwar Ansari, Fauzia Khan, Anees Ahmad

**Affiliations:** Industrial Chemistry Research Laboratory, Department of Chemistry, Aligarh Muslim University, Aligarh 202002, India

## Abstract

Cauliflower leaf powder (CLP), a biosorbent prepared from seasonal agricultural crop waste material, has been employed as a prospective adsorbent for the removal of a basic dye, methylene blue (MB) from aqueous solution by the batch adsorption method under varying conditions, namely, initial dye concentration, adsorbent dose, solution pH, and temperature. Characterization of the material by FTIR and SEM indicates the presence of functional groups and rough coarse surface suitable for the adsorption of methylene blue over it. Efforts were made to fit the isotherm data using Langmuir, Freundlich, and Temkin equation. The experimental data were best described by Freundlich isotherm model, with an adsorption capacity of 149.22 mg/g at room temperature. To evaluate the rate of methylene blue adsorption onto CLP, pseudo-first-order, pseudo-second-order, and intraparticle diffusion models were employed. The experimental data were best described by the pseudo-second-order kinetic model. Evaluation of thermodynamic parameters such as changes in enthalpy, entropy, and Gibbs' free energy showed the feasible, spontaneous, and exothermic nature of the adsorption process. On the basis of experimental results obtained, it may be concluded that the CLP prepared from agricultural waste has considerable potential as low-cost adsorbent in wastewater treatment for the removal of basic dye, MB.

## 1. Introduction

The increasing demand for commercial dyes by various industries leads to the vast production of dyes. Over 100000 commercial dyes are available and more than 7 × 10^5^ tons are produced annually, of which a significant portion is being discharged directly into the aqueous media [1, 2]. In developing nations, environmental pollution, particularly water pollution, which arises due to the discharge of unprocessed industrial effluents into main water streams is of major concern. These effluents containing dyes and pigments are regularly discharged into the natural water bodies by industries like food, textile, cosmetics, rubber, plastics, paper, pharmaceutical, and so forth. These dyes have so many adverse effects not only on the aquatic flora and fauna but also on the human health. Large water bodies can be colored even with small quantities of dyes, which not only affect visual quality but also diminish light penetration and photosynthesis. Many of these dyes are of toxic nature and have cancer-causing and mutagenic effects. So the effluents containing these coloring agents have to be removed appropriately earlier that they are discharged into the aquatic forms [3, 4]. Among these dyes, methylene blue (MB) is frequently used coloring substance for dying cotton, wood, and silk. Although methylene blue is not so harmful, it can cause harmful effect upon inhalation. Severe contact to methylene blue can cause increased heart rate, vomiting, tremor, Heinz body formation due to damage of hemoglobin component, cyanosis or blue disease, jaundice, quadriplegia, and tissue necrosis in humans [5], while ingestion through the mouth creates a hot feeling causing nausea, diarrhea, and gastric problems. Accidental large dose creates pain in abdomen and chest and head, abundant sweating, mental confusion, painful micturition, and methemoglobinemia [6]. This shows the necessity for effective removal of the dye from the effluent. During the previous year, several technologies comprising biological, chemical, and physical methods have been reported for the effective removal of dyes from polluted water [7, 8]. Among these approaches of dye removal, adsorption technique is one of the utmost operative methods for treatment of waste water in terms of cost, simple design, and easiness to operate. Adsorption is regarded as an easy, efficient, and economic process because it gives the best results with no harmful side products and therefore generates high quality treated effluents. Therefore, in recent years, this has prompted a growing research interest in using various conventional waste biomass by many researchers such as commercial activated carbons prepared by different activators, bioadsorbents, and wastes from agriculture such as coir pith, fruits peels, rice hull, saw dust, sugarcane bagasse, and wheat straw which have been used traditionally as an adsorbent for the removal of contagious pollutant [2, 9, 10]. In recent times, several approaches for the growth of economical and potent adsorbents have been studied. Various formal low-cost adsorbents including biosorbents, natural things, and waste materials from agriculture and industry have been suggested by a number of researchers for the removal of dyes from water. Some low-cost agricultural wastes like garlic peel [11], potato plant wastes [12], wild carrot (*Daucus carota*) [13], agricultural wastes-based activated carbons [14], cotton dust [15], chemically modified tea wastes [16], hazelnut husk activated carbon [17], rice husk and rice ash [18], tea wastes [19], fruit wastes (yellow passion) [20], plant stems (*Haloxylon recurvum*) [21], LDH bacteria aggregates [22], pine apple leaf powder [23], biomass (*Arthrospira platensis*) [24], and so forth have been efficiently used for the removal process but they suffer certain drawbacks as well.

Cauliflower, among the winter vegetables of the world, occupies an important place in the list. The crop is being grown across 3.8 million hectares in almost 150 diverse nations and produced around 70 million tons of cabbages annually. The major producer of this crop includes China, India, Spain, Italy, and France. The leaf of this vegetable is a waste product which can be gainfully utilized as a potential adsorbent for the elimination of methylene blue dye from waste water.

The main objective of the present study is to evaluate the potential of cauliflower leaf powder as an alternative adsorbent for the removal of methylene blue from aqueous media focusing on the adsorption capacity, optimization of various physicochemical parameters, and adsorption kinetics parameters. The equilibrium isotherms were described by various adsorption models and the isotherm constants were determined. In addition, the thermodynamic factors, such as change in enthalpy, entropy, and the free energy were also investigated in order to have a clear understanding of the reaction mechanisms and add a scientific basis to traditional uses of this vegetable.

## 2. Materials and Methods

### 2.1. Adsorbent: Cauliflower Leaf Powder (CLP)

Cauliflower leaf was brought from nearby agricultural field and thoroughly washed with tap water to remove dirt and adhering impurities from it. Then leaves were sun-dried to evaporate the moisture content. After that, it was further oven-dried for 24 h at 80°C to remove additional moisture content from it. Finally it was grinded into fine powder with the help of domestic mixer grinder and screened with molecular sieve of 100 mesh size (0.149 mm). Then, the organic impurities from this powder of 100 mesh particle size were removed by washing with double distilled water under vacuum pressure. The washed material was then dried overnight in an oven at 105°C. Finally dried material was grinded mechanically into fine powder with pastel and mortar and kept in desiccators for further use. No other physical or chemical treatments were performed prior to batch adsorption experiments.

### 2.2. Adsorbate: Methylene Blue

The basic dye methylene blue (MB), also known as Basic Blue 9, was obtained from Sigma-Aldrich and used as such without additional refining. The physical characteristics of the dye are listed in Table 1.

### 2.3. Adsorption Studies

Appropriate amount of MB dye (1 g) was dissolved in double distilled water to prepare a stock solution of methylene blue dye (1000 mg L^−1^); further desired concentration of adsorbate was prepared from the stock solution by diluting it. By varying one parameter under consideration and keeping other parameters constant, adsorbent dose, contact time, the effect of pH, initial dye concentration, and temperature on the adsorption of methylene blue dye on CLP were investigated. In each experiment, a preweighed amount of CLP was added to 20 mL of dye solution in a 100 mL conical flask and agitated at 90 rpm using a stirrer at room temperature for a given period of time. The dye concentration in supernatant solution was determined at wavelength of maximum absorption (*λ*_max_), that is, 664 nm, by double beam UV-visible spectrophotometer (PerkinElmer *λ*-25).

The amount of dye adsorbed (*q*_*e*_^exp^) and percentage dye adsorbed (%  *R*) on CLP were calculated by (1) and (2), receptively [25]:

(1) qeexp=Ci−Ce·Vw,

(2) % R=100·Ci−CeCi,

where *q*_*e*_^exp^ is quantity adsorbed on the adsorbent at equilibrium (mg g^−1^), *C*_*i*_ and *C*_*e*_ are the initial and equilibrium concentration (mg L^−1^) of dye in solution, respectively, *V* is the solution volume taken (L), and *w* is the mass of the adsorbent (g). All experiments were done three times and the mean values were used in data analysis.

### 2.4. Regeneration of the Adsorbent

For the frequent applications of used adsorbent (CLP), it was regenerated. The preweighed amount of used CLP was kept in 20 mL of 0.01 M different solvents such as H_2_SO_4_, HNO_3_, CH_3_COOH, and NaOH and was agitated for predetermined time interval and filtrate was measured spectrophotometrically. It was found that the adsorbent can be successfully regenerated with acidic solvents up to 6 successful cycles. After each cycle, adsorption capacity of the regenerated adsorbent gets reduced. The reduction in adsorption capacity was found from 164.23 mg g^−1^ to 111.25 mg g^−1^. Since adsorbent CLP used in this study for the MB dye removal is fairly inexpensive and freely obtainable, therefore regeneration does not seem to be certainly obligatory.

### 2.5. Error Analysis

In order to find most appropriate isotherm model for demonstrating the experimental data, chi square (*χ* ^2^), sum of squared error (SSE), sum of absolute error (SAE), and Marquardt's percent standard deviation (MPSD) error functions were applied and the model showing minimum error was calculated by the using following equation and is tabulated in Table 3.

Chi square, *χ* ^2^ is as follows:

(3) $$\begin{array}{c} \chi^{2}=\overset{n}{\underset{i=1}{\sum }}\left[\;\frac{q_{e}^{exp}-q_{e}^{cal}}{q_{e}^{cal}}\;\right]. \end{array}$$

## 3. Results and Discussion

### 3.1. Characterization of CLP

**Figure 1:**
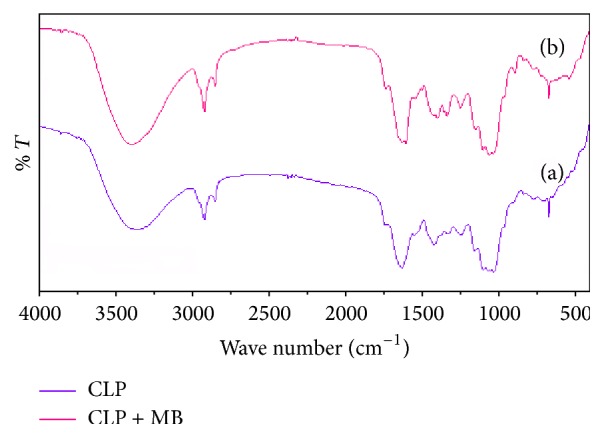
FT-IR spectra of CLP (a) before and (b) after the adsorption of methylene blue.

**Figure 2:**
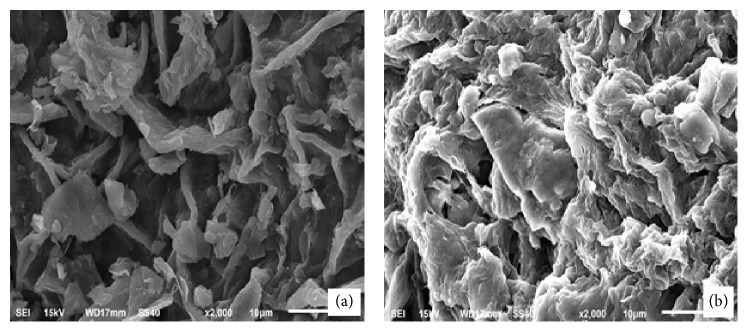
SEM image of CLP (a) before and (b) after the methylene blue adsorption.

**Figure 3:**
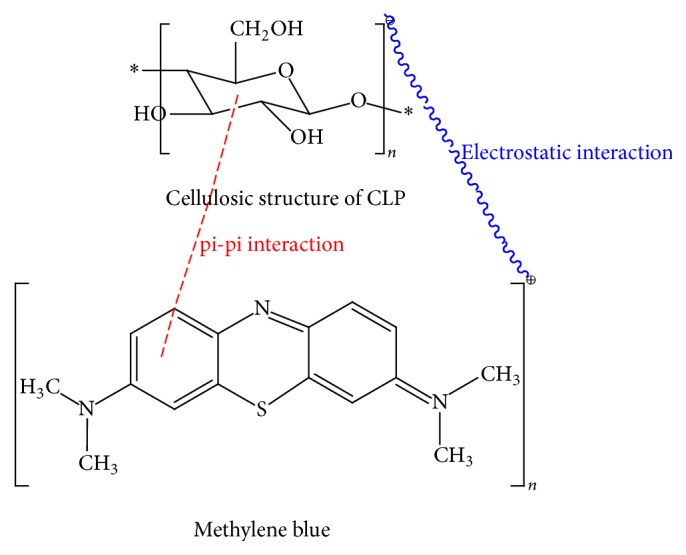
Proposed mechanisms for the adsorption of methylene blue dye over CLP.

The FTIR study was carried out using potassium bromide disc method over the wavelength region 4,000 cm^−1^–400 cm^−1^ to determine the presence of functional groups before and after the adsorption. The FTIR spectra were recorded on a FTIR spectrophotometer (PerkinElmer, version 10.4.00).  Figure 1(a) shows the FTIR spectrum of CLP before the adsorption of MB. The appearance of a broad peak at 3352 cm^−1^ is due to the stretching vibration of free -OH or -NH (str.) on the adsorbent surface as in pectin [26]. The peaks at 2921 cm^−1^ and 2851 cm^−1^ indicate the C-H stretching mode of aliphatic compounds. Peaks detected at 1738 cm^−1^, 1628 cm^−1^, 1418 cm^−1^, 1322 cm^−1^, and 1244 cm^−1^ correspond to C=O stretching, aromatic C=C bending, C-C stretching (in ring) aromatic, N-O symmetric stretch of nitro compounds, and C-N stretching of aliphatic amines, respectively. The peak at 1031 cm^−1^ might be due to C-O stretching of alcohols, carboxylic acids, esters, and ethers present on the surface of the biomass. The peaks around 1100–1000 cm^−1^ are known to be characteristics for all sugar derivatives [21].  Figure 1(b) shows the spectra of CLP after the adsorption of methylene blue dye. Considerable changes in the frequencies of functional groups were observed after the adsorption of MB dye at the surface of CLP due to their involvement in sorption process either through formation of chemical complex or through physical van der Waals forces [27]. The surface morphology of adsorbent was studied by Scanning Electron Microscopy (SEM).  Figure 2 shows the SEM micrograph of CLP at 2000x magnification before and after the adsorption of MB dye on its surface.  Figure 2(a) revealed that the surface of CLP was found to be coarse and asymmetrical and has significant numbers of pores which provides requisite sites for sorption of MB dye molecule.  Figure 2(b) showed that the MB molecules adsorbed at the surface of the adsorbent and therefore the morphology of the adsorbent surface has been changed significantly. A proposed mechanism of MB adsorption is shown in Figure 3.

### 3.2. Effect of pH

**Figure 4:**
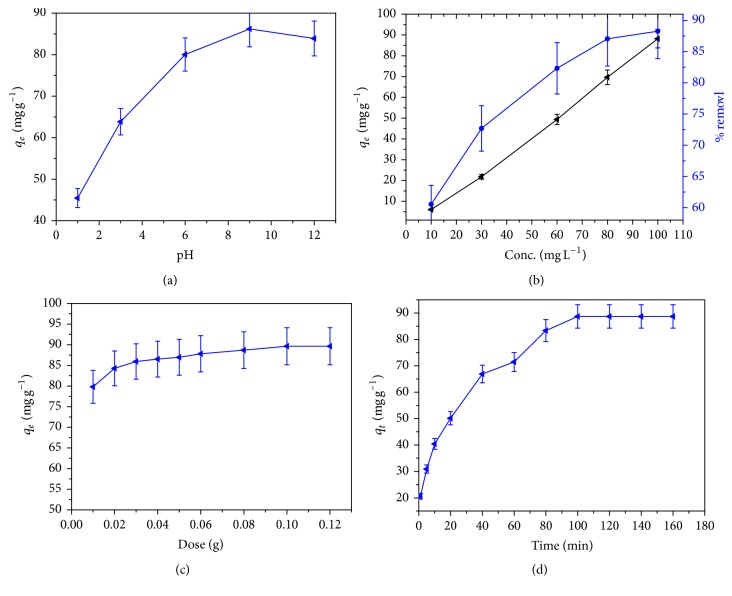
Adsorption studies for MB dye onto CLP (initial concentration: 100 ppm, mass of adsorbent = 0.02 g, volume of adsorbate = 20 mL, and agitation: 90 rpm, RT).

The pH of the solution controls the surface charge of the adsorbent and the degree of ionization of the pollutants. Since the effect of pH on the methylene blue adsorption on CLP was studied by varying the pH of dye solution over the range 1–10, the desired pH of the solution was adjusted by using 0.1 M HNO_3_ and 0.1 M NaOH solutions. To see the effect of pH, a series of experiments was done in a beaker containing 20 mL of dye solution and the desired pH was adjusted and 0.02 g CLP was added to the solution. The absorbance of the filtrate solution was taken at predetermined time. The biopolymers of biosorbent comprise many functional groups, so the net charge on the biosorbent is also pH dependent [28]. The number of negatively charged sites on the biosorbent increase as the pH of the system increases whereas the number of positively charged sites decreases, due to increase in the hydroxyl ion concentration [29].  Figure 4(a) showed the MB dye adsorption onto the biosorbent. The nature of the curve is in agreement with the fact that pH of the adsorption system plays an important role in the biosorptive removal of the MB dye. At lower pH, less number of negatively charge adsorbent cites and excess H^+^ cites are available at the CLP surface. Therefore, the lower sorption of MB at lower pH was probably due to the presence of the excess H^+^ ions competing with the cationic groups on the dye for sorption sites [30]. The maximum sorption of the MB (cationic or positively charged dye) dye was observed at pH 9 because, at higher pH, the surface of the CLP gets more negatively charged by losing protons (deprotonation of different functional groups on the surface of the biosorbent) and consequently supports the uptake of positively charged (cationic) dyes due to increased electrostatic force of attraction [31]. After that the decrease in the biosorption capacity of the CLP for MB decreases.

### 3.3. Effect of Concentration

To see the effect of concentration on the adsorption of methylene blue on CLP, different dye concentration over the range 10–200 mg per liter was tested with initial adsorbent dose 0.02 g. At predetermined time, the solution was centrifuged at 2000 rpm for 10 min and filtrate was taken out with the help of syringe. The absorbance of the solution was measured spectrophotometrically. It can be seen from Figure 4(b) that as the concentration of adsorbate solution increases, the corresponding adsorption capacity also increases and reaches maximum at a point where the adsorption remains constant. There is no further improvement in adsorptive removal after that concentration. It may be concluded that the adsorption of MB at the surface of CLP is highly dependent on the initial concentration of the MB dye in solution and it increases, reaching up to a maximum value, while % removal of MB decreases as its initial concentration increases.

### 3.4. Effect of Adsorbent Dose

The amount of adsorbent plays an important role in the adsorption of MB dye from the aqueous solution. The plot between quantities of dye adsorbed *q*_*e*_ against dose of adsorbent is shown in Figure 4(c). It is very clear from the plot that quantity of dye adsorbed is varied with varying adsorbent weight and it decreased with increasing adsorbent weight. The quantity of MB dye adsorbed drops from 149.2 mg per gram to 17.74 mg per gram when the adsorbent dose increases from 0.01 g to 0.1 g (not plotted), whereas % removal increased from 74.81 to 88.1 for the same increment of adsorbent dose. At higher CLP to MB concentration ratio, there is a very fast superficial sorption onto the adsorbent surface that produces a lower solute (MB) concentration in the solution. When the CLP to MB concentration ration is lower, the superficial sorption onto the adsorbent surface is slow so higher concentration of solute (MB) remains in the solution. The reason for this may be due to fact that a fixed amount of CLP adsorbent can only adsorb a certain amount of MB dye. The decrease in quantity adsorbed at the surface of the CLP adsorbent (*q*_*e*_) with increase in adsorbent dose is due to the concentration gradient or split in the flux between MB dye concentration in the solution and the MB concentration in the surface of CLP. Therefore, the amount of dye adsorbed onto unit mass of adsorbent decreased with increasing adsorbent weight, thus causing a fall in amount of dye adsorbed (*q*_*e*_) value with increasing the mass of adsorbent [32].

### 3.5. Effect of Ionic Strength

**Figure 5:**
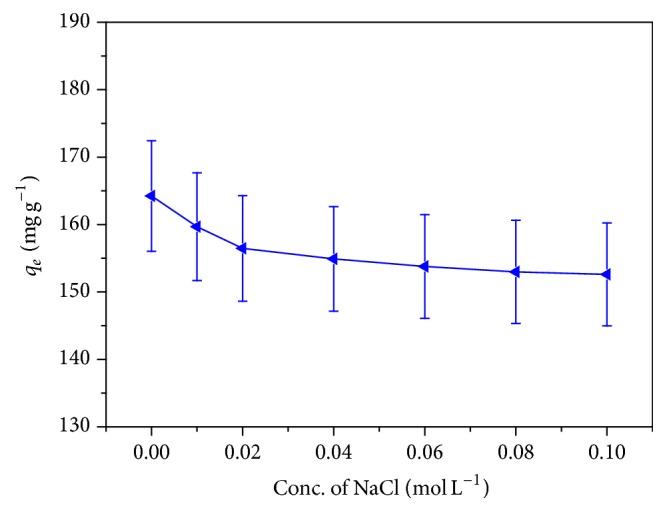
Effect of ionic strength on the adsorption of methylene blue over CLP (concentration: 100 mg L^−1^, temperature: 303 K, and dose: 0.1 g).

The adsorption capacity of an adsorbent is generally affected by high ionic strength present in waste water, attributed by the existence of salt in higher concentration. In order to see the effect of the salt on the adsorption behavior of an adsorbent, different concentration of NaCl over the range 0.0–0.1 M was studied.  Figure 5 shows contrary effect of ionic strength on the adsorption of MB dye at the surface of CLP. As the concentration of salt increases, the quantity of MB dye adsorbed at the surface of the CLP decreases and reaches constant value after which no reduction in the adsorption capacity of the CLP was observed. This behavior of decreasing adsorbed amount of dye at the surface of CLP with increasing the NaCl concentration is due to the fact that salt screens the electrostatic interaction between the opposite charges of the adsorbent surface and the dye molecule [33].

### 3.6. Adsorption Kinetic Studies

**Figure 6:**
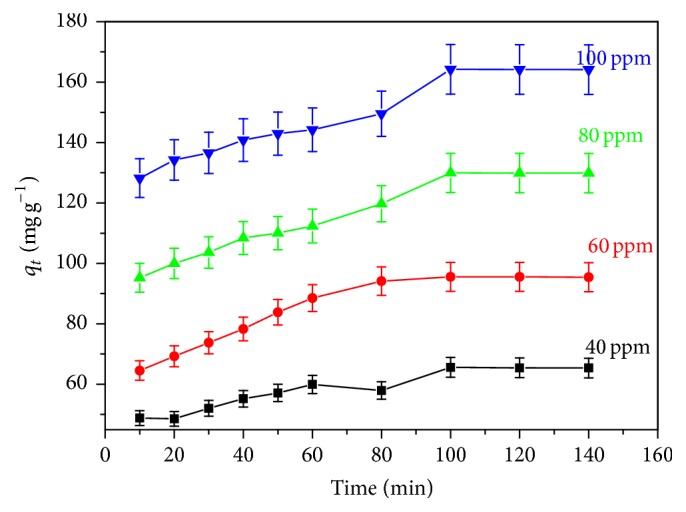
Effect of contact time on MB adsorption onto CLP.

In order to efficaciously use agricultural by product as a potential adsorbent, contact time is of fundamental importance [34]. The equilibrium time specifies the promising diffusion control mechanism of the sorbet as it moves toward the sorption surface [35]. The effects of contact time on adsorption of methylene blue onto CLP were studied under predetermined experimental condition for 100 mg L^−1^ at 303, 313, and 323 K. It can be seen from Figures 4(d) and 6 that the rate of adsorption was very rapid at initial period of contact time and as the contact time increases, the adsorption capacities also increase. It reaches a maximum value after which there is no increment in adsorption capacity of the adsorbent. The rapid rise in the slope of the curve pointed to the rapid binding of the MB dye with the CLP surface, that is, rapid adsorption region. This may be attributed due to greater number of vacant sites available on CLP adsorbent and strong attractive forces between the dye molecule and the adsorbent [21, 36]. This rapid increase in adsorption capacities during initial stage showed that the nature of binding was a physical one [37]. As the contact time increases further, the number of vacant sites also decreases resulting in the decreased rate of dye binding [38]. The equilibrium time for adsorption of MB dye at the surface of CLP was found to be 100 min and thereafter there is no considerable change in the adsorption capacities of CLP adsorbent.

The adsorption kinetics of the methylene blue solution at the surface of CLP was studied for the concentration of 40, 60, 80, and 100 mg L^−1^ and data were applied to Lagergren pseudo-first-order (3), Ho's pseudo-second-order (4) and intraparticle diffusion model (5) [39, 40]. Lagergren pseudo-first-order kinetics model, which is particularly suitable for low concentrations, indicates that the process of sorption occurs at a rate proportional to the dye concentration [39]. The second-order kinetics in which the rate controlling step is an exchange reaction is thought to derive from sorption processes [39].

(4) ln⁡qe−qt=ln⁡qe−K1t,

(5) tqt=1K2qe2+tqe,

(6) qt=Kit1/2+C.

In the above-mentioned equations, *q*_*e*_ (mg L^−1^) and *q*_*t*_ (mg L^−1^) are the amounts of MB dye adsorbed at equilibrium and at any contact time of adsorption *t* (min.), respectively; *K*_1_ (min.^−1^), *K*_2_ (g mg^−1^ min^−1^), and *K*_*i*_ (mg g^−1^ min.^−1*/*2^) are the pseudo-first-order, pseudo-second-order, and intraparticle diffusion rate constant, respectively.

**Figure 7:**
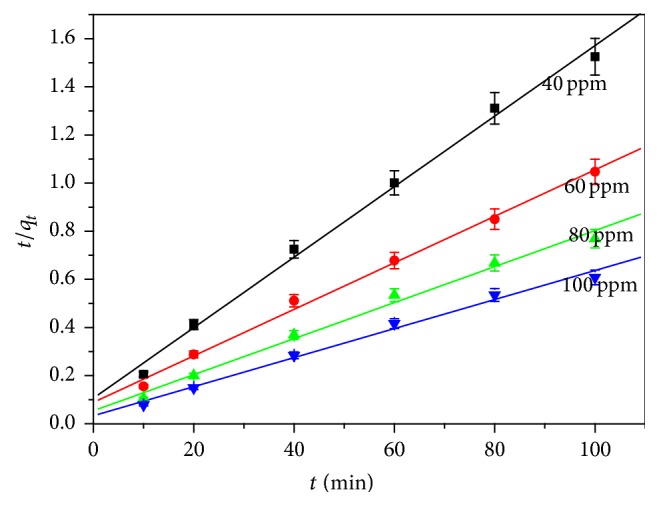
Pseudo-second-order kinetic plots for the adsorption of MB onto CLP (dose: 0.1 g, temperature: 303 K).

Accordingly, the experimental data were fitted to these kinetics models. The slopes and intercepts were calculated from the equation of straight line obtained. The data obtained along with *R* ^2^ values are presented in Table 2. The sorption system will follow a specific kinetic model if *R* ^2^ value exceeds 0.98 and *q*_*e*_^cal^ value is close to that of the experimental value [37]. On the basis of *R* ^2^ values, Ho's pseudo-second-order kinetic model was proficient to explain the kinetics of the process (Figure 7). The values of *q*_*e*_^exp^ and *q*_*e*_^cal^ were relatively closer for Ho's pseudo-second-order model rather than the Lagergren pseudo-first-order model. This is in accordance with the previous studies that Ho's pseudo-second-order kinetic model was followed in biosorption by agricultural materials [21]. From Table 2, in the case of pseudo-second-order kinetic model it is very clear that as the initial concentration of MB increases, the value of *q*_*e*_^exp^ also increases because, at high concentration, the great competition for the vacant sites leads to the higher sorption rates [41]. There are four consecutive steps used to describe the mechanism of adsorption process. These are as follow:

1. Adsorbate ions are transported to the liquid film or boundary layer from bulk liquid, surrounding the adsorbent
2. Adsorbate ions are transported from boundary film to external surface of the adsorbent during the surface diffusion phenomenon
3. Adsorbate ions are transferred from the surface to the intraparticle active sites of the adsorbent during the pore-diffusion phenomenon
4. There are sorption and desorption that take place within the particle and on the external surface

The first and fourth steps do not belong to the rate controlling steps because in the first step there is no involvement of adsorbent and the fourth step is a very rapid process. Therefore, surface or pore-diffusion may be the rate controlling steps [42]. Weber and Morris' model is widely used to predict the rate controlling step [40]. The calculated intraparticle diffusion model parameters for methylene blue adsorption onto the CLP are listed in Table 3. On the basis of result obtained, it may be concluded that intraparticle diffusion is not the rate controlling mechanism for the adsorption of MB dye from aqueous solution on the surface of CLP [41].

### 3.7. Adsorption Isotherms

**Figure 8:**
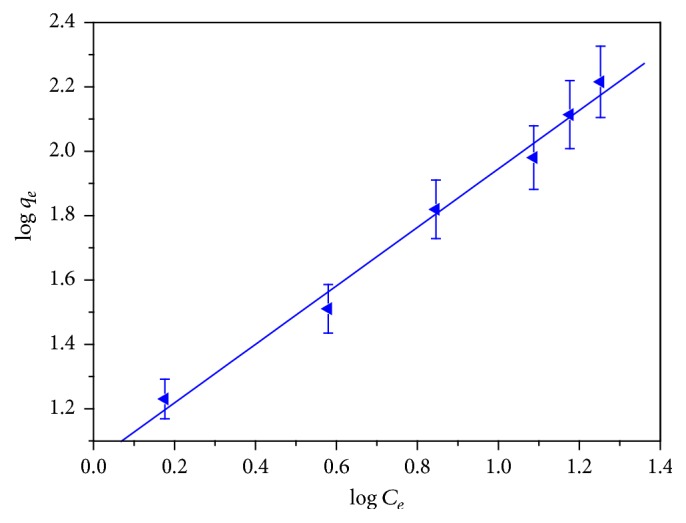
Freundlich isotherm for the adsorption of MB onto CLP.

The adsorption isotherms perform significant role in the design of any adsorption system. In this study Langmuir, Freundlich, and Temkin adsorption isotherm models were used to describe the distribution of adsorbate molecule between the solid phase and the liquid phase at the attainment of adsorption equilibrium. The experiment of the adsorption isotherms was conducted by adding 0.1 g of adsorbent dose into 20 mL of adsorbate solution at room temperature for predetermined time. The adsorption capacity of the adsorbent was calculated by using (1). Since the experimental data is obtained when applied to these isotherm models, it does not follow the Langmuir and Temkin models, so these were omitted. According to Freundlich isotherm model, adsorption takes place on heterogeneous surfaces and there is interaction between adsorbate molecules adsorbed that gives infinite surface coverage. The equation used to describe the Freundlich isotherm can be written as

(7) $$\begin{array}{c} q_{e}=K_{F}\cdot C_{e}^{1/n}, \end{array}$$

where *q*_*e*_ is the quantity of methylene blue adsorbed per g of adsorbent (mg g^−1^) and *C*_*e*_ is the equilibrium concentration of methylene blue in the solution (mg L^−1^). The Freundlich constant *K*_*F*_ is the maximum multilayer adsorption capacity and *n* is the adsorption intensity [43]. The Logarithm of both sides of (7) expresses the linear form of the Freundlich isotherm, and Figure 8 is a plot log *q*_*e*_ versus log *C*_*e*_ used to obtain the values of *K*_*F*_ and 1/*n*.

(8) $$\begin{array}{c} \log q_{e}=\log K_{F}+\frac{1}{n}\log C_{e}. \end{array}$$

The heterogeneity factor *n* can be used to indicate whether the adsorption is linear (*n* = 1), a chemical process (*n* < 1), or a physical process (*n* > 1). In the present study, Table 3 shows that value of the heterogeneity factor is greater than 1 (*n* > 1) indicating that the adsorption of MB dye at the surface of CLP is physical in nature. The high *K*_*F*_ value gives the adsorption of aggregated molecule which is favorable at 303 K. The calculated adsorption capacity (*q*_*e*_^cal^) was found to be 149.22 mg g^−1^ that is very close to the experimental adsorption capacity (*q*_*e*_^exp^) having the *R* ^2^ value near 1 and minimum chi square error (*χ* ^2^).

### 3.8. Thermodynamic Studies: Effect of Temperature

To see the effect of temperature on the adsorption of methylene blue dye on to the surface of CLP, a series of experiments were carried out at 303 K, 313 K, and 323 K. It was found that the adsorption capacity decreased from 164.23 mg g^−1^ to 149.58 mg g^−1^ as the temperature of system was increased from 303 K to 323 K. This trend may be due to the affinity of the dye molecules to outflow to the bulk phase from the solid phase with an increase in temperature of the solution [33]. The thermodynamic parameters were calculated by using the following equations:

(9) Kd=qeCe,

(10) ΔG0=−RTln⁡Kd,

(11) ln⁡Kd=ΔS0R−ΔH0RT,

where *K*_*d*_ is distribution coefficient, *R* is the universal gas constant (J mol^−1^ K^−1^), *T* is absolute temperature of the solution, and Δ*G* ^0^, Δ*S* ^0^, and Δ*H* ^0^ are Gibb's free energy (kJ mol^−1^), Change in entropy (kJ mol^−1^ K), and change in enthalpy (kJ mol^−1^), respectively [37].

**Figure 9:**
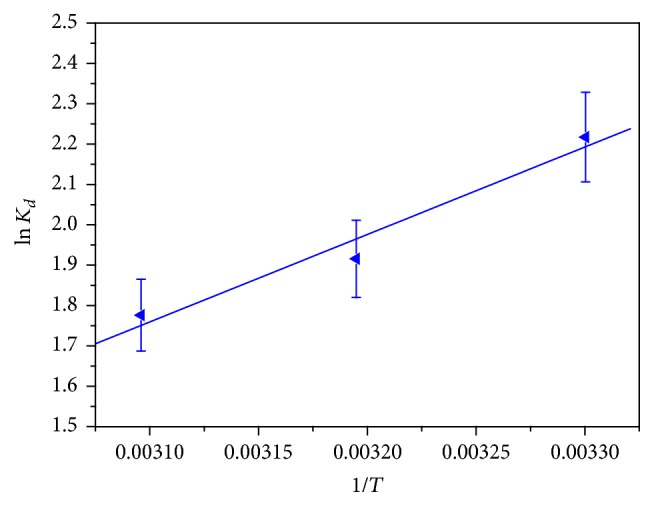
Plot of ln⁡*K*_*d*_ versus 1/*T* for the adsorption of MB onto CLP.

**Figure 10:**
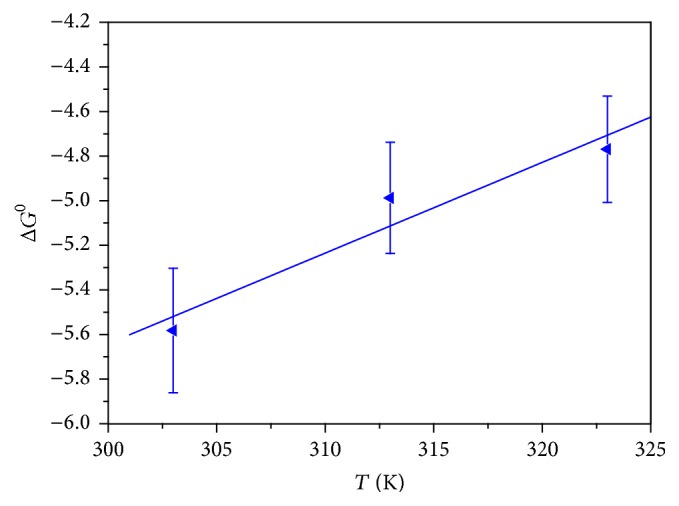
Plot of Δ*G* ^0^ versus *T* for the adsorption of MB onto CLP.

The slopes (−Δ*H* ^0^/*R*) and intercepts (Δ*S* ^0^/*R*) of the Figure 9 give the values of Δ*S* ^0^ and Δ*H* ^0^. The Δ*G* ^0^ values were calculated using (10).  Figure 10 showed a plot of Δ*G* ^0^ versus *T* yielding a straight line. The values of thermodynamics parameters obtained are summarized in Table 4. The negative value of Gibb's free energy change showed that the adsorption of MB dye at the surface of the CLP was feasible and spontaneous in nature. The negative value of Δ*S* ^0^ and Δ*H* ^0^ shows the decrease in the randomness at the CLP-MB interface during the adsorption and exothermic nature for the overall process, respectively. Further, Δ*H* ^0^ < 40 kJ/mol shows that the nature of the sorption process was physisorption [33, 44].

## 4. Conclusion

The current study revealed that the cauliflower leaf powder, an agricultural waste produced after harvesting the crop, can be used as an adsorbent for the removal of methylene blue dye from synthetic aqueous solutions. The quantity of dye adsorbed was found to vary with initial solution concentration, pH, adsorbent dose, and contact time. The quantity of dye uptake was found to increase with increase in initial concentration of dye solution and contact time and found to decrease with increase in adsorbent dosage but the percent removal increases. The sorption data were found to follow pseudo-second-order kinetics since amounts of methylene blue dye obtained experimentally, that is, 164.23 mg g^−1^, and calculated, that is, 166.66 mg g^−1^, from the plot are close to one another having regression value 0.991. Equilibrium data were fitted very well in Freundlich isotherm equation approving the sorption capacity of methylene blue onto cauliflower leaf powder with a sorption capacity of 149.22 mg g^−1^ having regression value 0.989 and minimum chi square error 1.5 showing the suitability of this model to describe the adsorption of MB dye onto CLP. The negative values of Δ*G* ^0^ and Δ*H* ^0^ indicate that the adsorption process is spontaneous, feasible, and exothermic in nature.

## Supplementary Material

The suplimentary material, cauliflower belongs to the family Brassicaceae is one of several vegetables in the species Brassica oleracea. This crop is an annual plant that reproduces by seed. In general, edible part is only the head (the white curd) and its leaves being discarded as waste.

## Figures and Tables

**Table 1:** Physical and chemical properties of adsorbate (methylene blue).

Properties	Values
Chemical structure	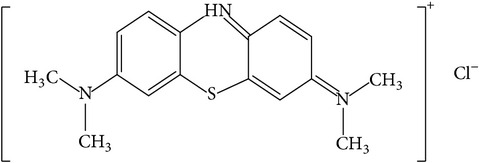
Chemical formula	C_16_H_18_N_3_SCl
Wave length	664 nm
IUPAC name	3,7-Bis(dimethylamino)-phenothiazin-5-ium chloride
Molecular weight	319.85 (g mol^−1^)
Type	Cationic dye
Color	Blue
Solution pH	6.5
pH_ZPC_	6.23
Solubility	Soluble in water

**(a):** 

*C*_*i*_ (mg L^−1^)	Pseudo-first-order kinetic parameters			
	*q*_*e*_^exp^ (mg g^−1^)	*q*_*e*_^cal^ (mg g^−1^)	*K*_1_	*R* ^2^
40	65.573	22.780	0.021	0.918
60	95.554	63.351	0.0423	0.965
80	129.958	42.093	0.0166	0.974
100	164.230	40.021	0.0124	0.978

**(b):** 

*C*_*i*_ (mg L^−1^)	Pseudo-second-order kinetic parameters			
	*q*_*e*_^exp^ (mg g^−1^)	*q*_*e*_^cal^ (mg g^−1^)	*K*_2_	*R* ^2^
40	65.573	68.027	0.0021	0.994
60	95.554	103.093	0.0012	0.995
80	129.958	133.333	0.0010	0.990
100	164.230	166.667	0.0011	0.991

**(c):** 

*C*_*i*_ (mg L^−1^)	Intraparticle diffusion parameters		
	*K*_*i*_ (mg g^−1^ min.^−1/2^)	*C*	*R* ^2^
40	2.578	39.03	0.961
60	4.927	48.31	0.983
80	4.765	78.66	0.965
100	4.578	112.5	0.914

**Table 3:** Adsorption isotherm parameters for adsorption of MB on CLP.

Freundlich parameters				
*q*_*e*_^cal^ (mg g^−1^)	*K*_*F*_	*n*	*R* ^2^	χ^2^ (chi square)
149.22	10.879	1.101	0.989	1.509

**Table 4:** Thermodynamic parameters for adsorption of MB on CLP.

Adsorption system	Δ*H* ^0^ (kJ mol^−1^)	Δ*S* ^0^ (kJ mol^−1^ K^−1^)	Δ*G* ^0^ (kJ mol^−1^)		
			303 K	313 K	323 K
CLP-MB	−18.015	−0.041	−5.531	−5.119	−4.707
