# Supplementary material for: Cauliflower Leave, an Agricultural Waste Biomass Adsorbent, and Its Application for the Removal of MB Dye from Aqueous Solution: Equilibrium, Kinetics, and Thermodynamic Studies
Source: Int J Anal Chem. 2016 Nov 15;2016:8252354. doi: 10.1155/2016/8252354 (PMC5126461; doi:10.1155/2016/8252354)
Supplement: Supplementary file 1 — The suplimentary material, cauliflower belongs to the family Brassicaceae is one of several vegetables in the species Brassica oleracea. This crop is an annual plant that reproduces by seed. In general, edible part is only the head (the white curd) and its leaves being discarded as waste. [file 8252354.f1.docx]

Graphical Abstract
